# Editorial: Cardiomyopathy and heart failure in oncology

**DOI:** 10.3389/fonc.2026.1829234

**Published:** 2026-04-22

**Authors:** Marta Adamiak, Anna Borowiec, Anuradha Kalyanasundaram, Prabhu Mathiyalagan

**Affiliations:** 1 Benthos Prime Central, Houston, TX, United States; 2 Maria Sklodowska-Curie National Research Institute of Oncology, Warsaw, Poland; 3 University of North Texas Health, Fort Worth, TX, United States

**Keywords:** cancer survivorship, cancer therapy-related cardiotoxicity, cardiomyopathy, cardio-oncology, heart failure, precision prevention, artificial intelligence, AI in Medicine

## 1 Introduction

Advances in cancer therapy have transformed malignancies once considered fatal into chronic and often curable conditions (1). However, this success has been accompanied by a growing burden of cardiovascular disease, particularly cardiomyopathy and heart failure, which now represent major determinants of long-term morbidity and mortality in cancer survivors (2, 3). Cancer therapies exert complex myocardial, vascular, metabolic, inflammatory, and electrical effects that may manifest acutely or decades after treatment completion (4). As survivorship expands, the integration of cardiovascular prevention, early detection, and management strategies into oncologic care has become an essential component of comprehensive cancer medicine (5).

This Research Topic, *Cardiomyopathy and Heart Failure in Oncology*, brings together 13 contributions spanning mechanistic insights, advanced diagnostics, clinical case reports, preventive strategies, emerging therapies, and digital innovation. Collectively, these studies reflect the multidimensional nature of cardio-oncology and emphasize that cardiomyopathy and heart failure in cancer patients are not isolated phenomena but part of a broader continuum of myocardial and vascular injury, arrhythmogenesis, and systemic metabolic dysfunction. The contributions can be broadly categorized into five interrelated domains as follows:

## 2 Treatment-related cardiotoxicity and long-term survivorship

Understanding long-term cardiovascular risk in cancer survivors is fundamental to preventing late-onset cardiomyopathy and heart failure. Orszaghova et al. provide a comprehensive review of cardiovascular toxicity in survivors of testicular germ cell tumors (TGCT), a population characterized by excellent oncologic outcomes but substantial long-term cardiovascular burden. Cisplatin-based chemotherapy, a cornerstone of TGCT treatment, is associated with an increased risk of myocardial infarction, cerebrovascular events, thromboembolism, and heart failure, with risk peaking early after treatment yet persisting for decades. The review highlights vascular injury mechanisms, including endothelial dysfunction, oxidative stress, inflammation, and prothrombotic states, as well as myocardial damage pathways leading to cardiomyopathy. Importantly, the authors underscore the high prevalence of traditional cardiovascular risk factors among TGCT survivors, emphasizing the need for structured survivorship care models integrating cardiovascular risk stratification, aggressive risk factor modification, and lifelong monitoring.

Complementing this survivorship perspective, Trabattoni et al. (CLARIFIER study) examine coronary artery calcium (CAC) and cardiovascular risk profiles in women at least five years after breast radiotherapy. Notably, CAC burden correlated strongly with traditional cardiovascular risk factors, including age, smoking, hypertension, dyslipidemia, and early menopause, rather than radiation dose or laterality, reinforcing the central role of baseline cardiometabolic risk in shaping long-term cardiovascular outcomes after cancer therapy. These findings suggest that cardiomyopathy and ischemic heart disease risk after radiotherapy may be modifiable through early preventive strategies and targeted cardiovascular risk management, rather than being determined solely by radiation exposure.

Together, these studies highlight a paradigm shift from viewing cardiotoxicity as a rare complication to recognizing it as a predictable and modifiable determinant of long-term outcomes.

## 3 Acute and atypical cardiomyopathy phenotypes in oncology

Several contributions illuminate the heterogeneity of cardiomyopathy phenotypes encountered in oncology, extending beyond classical chemotherapy-induced systolic dysfunction. Potievskaya et al. report a case of Takotsubo cardiomyopathy triggered by acute surgical stress and severe systemic inflammation in a young woman with colorectal cancer complicated by intestinal necrosis and peritonitis. The patient developed acute heart failure with markedly reduced left ventricular ejection fraction and ECG changes mimicking myocardial infarction, despite unobstructed coronary arteries, with subsequent full recovery. This case underscores the susceptibility of oncology patients to stress-mediated cardiomyopathy in the setting of acute illness, surgery, and inflammatory states, and highlights the importance of recognizing reversible cardiomyopathy phenotypes in cancer populations.

Similarly, Szmit et al. describe a dramatic case of capecitabine-associated cardiotoxicity presenting as ST-segment elevation myocardial infarction with severe left ventricular dysfunction, malignant ventricular arrhythmias, and cardiac arrest, in the absence of obstructive coronary disease. Transient coronary vasospasm was deemed the most likely mechanism, and the patient ultimately required wearable defibrillator therapy and subsequent implantable cardioverter–defibrillator implantation. This case illustrates the life-threatening potential of fluoropyrimidine cardiotoxicity and its capacity to produce acute ischemic and arrhythmogenic phenotypes that may progress to cardiomyopathy and heart failure, even when coronary arteries are angiographically normal.

Xie et al. expand the spectrum of oncologic cardiac disease by reporting a rare case of primary cardiac lymphoma presenting as a right atrial mass initially misdiagnosed as thrombus or myxoma. Cardiac MRI revealed infiltrative features with characteristic enhancement patterns, but definitive diagnosis required histopathological confirmation. Despite surgical resection, the patient declined chemotherapy and experienced rapid clinical deterioration. While not a classical cardiomyopathy, this case highlights how primary cardiac malignancies may directly impair myocardial function and hemodynamics, contributing to heart failure syndromes and underscoring the importance of advanced imaging and tissue diagnosis in atypical cardiac presentations in oncology.

Collectively, these case-based contributions demonstrate that cardiomyopathy in cancer patients encompasses a wide range of phenotypes, including stress-induced, ischemic, inflammatory, arrhythmogenic, and infiltrative, each requiring distinct diagnostic and therapeutic approaches.

## 4 Advanced diagnostic and monitoring strategies for early detection

Early identification of subclinical myocardial injury before the development of overt cardiomyopathy or heart failure represents a central goal in cardio-oncology. Oksen et al. provide original data demonstrating that combined echocardiographic global longitudinal strain (GLS) and ECG repolarization markers can detect early myocardial dysfunction in patients receiving immune checkpoint inhibitors (ICIs). In their cohort, GLS declined significantly over six months, accompanied by increased QT dispersion, QTc prolongation, Tp–e interval prolongation, and heart rate elevation, even in the absence of overt left ventricular ejection fraction decline. The strong correlation between GLS and repolarization abnormalities supports the concept that mechanical and electrical myocardial remodeling may occur in parallel during early cardiotoxicity, offering complementary diagnostic signals for surveillance strategies.

Extending this concept, Sonaglioni et al. present a systematic review of imaging-based assessments of biventricular mechanics during ICI therapy. Across 12 studies and 554 patients, conventional echocardiographic measures such as ejection fraction remained largely unchanged, while strain-based parameters, including LV-GLS, circumferential and radial strain, RV-GLS, and atrial strain, declined early, signaling subclinical myocardial dysfunction. Cancer therapy–related cardiac dysfunction occurred in approximately one-third of patients, whereas myocarditis remained rare but potentially fatal. This review reinforces the superiority of strain imaging over traditional metrics for detecting early cardiotoxicity and suggests that biventricular and atrial dysfunction may precede overt heart failure.

Beyond myocardial mechanics, Zhang et al. investigated atrial arrhythmias in cancer patients with coronary heart disease highlights the importance of rhythm surveillance as a component of cardiomyopathy risk stratification. In this cohort, atrial arrhythmias occurred predominantly in patients with pre-existing coronary heart disease, with smoking, ischemic heart disease, and reduced left ventricular ejection fraction emerging as independent predictors. These findings emphasize that electrical remodeling and arrhythmogenesis represent parallel pathways to heart failure development in oncology patients, particularly among those with underlying cardiovascular disease.

## 5 Cardioprotective therapies and preventive strategies

Uzun et al. contribute compelling preclinical evidence that dapagliflozin, a sodium-glucose cotransporter 2 (SGLT2) inhibitor, mitigates acute 5-fluorouracil–induced cardiotoxicity in a rat model. Dapagliflozin preserved left ventricular ejection fraction, reduced ST-segment elevation and arrhythmogenic ECG changes, and attenuated myocardial inflammation and tissue injury. These findings extend the cardioprotective profile of SGLT2 inhibitors beyond heart failure populations and suggest a potential role in mitigating chemotherapy-induced myocardial injury, warranting further translational and clinical investigation.

These experimental findings align closely with the systematic review by Sonaglioni et al. on SGLT2 inhibitors as emerging cardioprotective agents in anthracycline-treated populations. Across 52 studies, SGLT2 inhibitors demonstrated consistent mechanistic and functional cardioprotection in doxorubicin models, with supportive signals in observational oncology cohorts. In contrast, traditional cardioprotective strategies, such as ACE inhibitors and beta-blockers, exhibited heterogeneous benefits and were often limited to surrogate endpoints. The authors further highlight the lack of randomized head-to-head trials and call for standardized prospective studies.

Complementing these emerging pharmacologic approaches, Migliari et al. provide a comprehensive review on prevention of cancer therapy–related cardiotoxicity synthesizes current evidence for pharmacological and non-pharmacological strategies across the cardio-oncology continuum. The authors discuss contemporary definitions of cardiotoxicity, emphasize early detection through biomarkers and advanced imaging, and evaluate evidence supporting RAAS inhibitors, beta-blockers, dexrazoxane, statins, SGLT2 inhibitors, and angiotensin receptor–neprilysin inhibitors. They further highlight the importance of lifestyle interventions, exercise, nutrition, psychological support, and multidisciplinary care models, as well as the growing role of telemedicine and digital health tools.

## 6 Emerging technologies and precision cardio-oncology

Segura et al. provide a forward-looking perspective on the role of artificial intelligence (AI) in cardio-oncology, emphasizing its potential to transform risk stratification, early detection, and personalized prevention of cardiomyopathy and heart failure. AI-based applications in ECG, echocardiography, cardiac MRI, biomarkers, and wearable devices have demonstrated promising performance in predicting chemotherapy-related cardiac dysfunction and identifying early myocardial injury before overt clinical deterioration. In particular, AI-derived ECG signatures have shown superior predictive capacity compared with traditional risk models in anthracycline-treated patients, while automated imaging analysis enhances reproducibility and efficiency of ventricular function assessment.

However, the authors appropriately highlight substantial barriers to widespread clinical adoption, including lack of external validation, algorithmic bias, data privacy concerns, limited interpretability, and regulatory challenges.

## 7 Integrating evidence across the cardio-oncology continuum

Studies highlight that cardiotoxicity is not limited to systolic dysfunction but encompasses stress cardiomyopathy, arrhythmogenic syndromes, ischemic mimics, infiltrative disease, and subclinical myocardial remodeling detectable only through advanced diagnostics. In this context, Li et al. provide mechanistic evidence that radiotherapy-induced myocardial injury can be attenuated while preserving antitumor efficacy, illustrating the potential for targeted interventions that decouple oncologic benefit from cardiovascular harm.

The collective evidence emphasizes four overarching priorities for the field. First, early detection must move beyond left ventricular ejection fraction toward multimodal surveillance strategies incorporating strain imaging, ECG repolarization markers, biomarkers, and digital health tools. Second, prevention strategies must evolve from empiric cardioprotective therapy toward precision approaches informed by mechanistic insights, pharmacogenomics, and AI-driven risk stratification. Third, survivorship care must integrate long-term cardiovascular monitoring and aggressive risk factor modification into routine oncology practice, particularly for populations exposed to highly cardiotoxic regimens such as anthracyclines, cisplatin, fluoropyrimidines, and immune checkpoint inhibitors. Fourth, multidisciplinary collaboration across cardiology, oncology, imaging, digital health, and basic science remains essential to translating mechanistic discoveries into clinically meaningful improvements in patient outcomes.

## 8 Conclusions and future directions

This Research Topic reflects the rapidly evolving landscape of cardio-oncology and underscores that cardiomyopathy and heart failure are no longer rare complications of cancer therapy but predictable and, increasingly, preventable consequences of modern oncologic care.

Future research priorities include the development of standardized definitions and endpoints for cardiotoxicity and cardiomyopathy, large-scale prospective trials of cardioprotective therapies, particularly SGLT2 inhibitors and novel agents, and validation of AI-driven risk stratification tools across diverse populations. Equally important is the implementation of multidisciplinary survivorship programs that embed cardiovascular prevention into routine cancer care pathways.

By bringing together experimental studies, clinical investigations, systematic reviews, and conceptual frameworks, this Research Topic advances the field toward a more comprehensive, mechanistically informed, and patient-centered approach to cardiomyopathy and heart failure in oncology. Ultimately, the goal is not merely to preserve cardiac function but to ensure that the remarkable gains in cancer survival are matched by equally durable cardiovascular health and quality of life.
